# Expression and Cellular Localization of 15-Hydroxy-Prostaglandin-Dehydrogenase in Abdominal Aortic Aneurysm

**DOI:** 10.1371/journal.pone.0136201

**Published:** 2015-08-19

**Authors:** David Solà-Villà, Jaime-Félix Dilmé, Cristina Rodríguez, Begoña Soto, Luis Vila, José-Román Escudero, José Martínez-González, Mercedes Camacho

**Affiliations:** 1 Autonomous University of Barcelona, Institute of Biomedical Research (II-B Sant Pau), Barcelona, Spain; 2 Angiology, Vascular Biology and Inflammation Laboratory, Institute of Biomedical Research (II-B Sant Pau), Barcelona, Spain; 3 Vascular Surgery Department, Institute of Biomedical Research (II-B Sant Pau), Barcelona, Spain; 4 Cardiovascular Research Center (CSIC-ICCC), Institute of Biomedical Research (II-B Sant Pau), Barcelona, Spain; Max-Delbrück Center for Molecular Medicine (MDC), GERMANY

## Abstract

PGE_2_ has been implicated in abdominal aortic aneurysm (AAA) associated hypervascularization. PGE_2_-metabolism involves 15-hydroxyprostaglandin-dehydrogenase (15-PGDH) the expression of which in AAA is unknown. The aim of this study was to examine the expression and cell distribution of 15-PGDH in AAA. Here, we show that 15-PGDH mRNA levels were significantly higher in aorta samples from patients undergoing AAA repair than in those from healthy multiorgan donors. Consequently, the ratio of metabolized PGE_2_ secreted by aortic samples was significantly higher in AAA. AAA production of total PGE_2_ and PGE_2_ metabolites correlated positively with PGI_2_ production, while the percentage of metabolized PGE_2_ correlated negatively with the total amount of PGE_2_ and with PGI_2_. Transcript levels of 15-PGDH were statistically associated with leukocyte markers but did not correlate with microvascular endothelial cell markers. Immunohistochemistry revealed 15-PGDH in the areas of leukocyte infiltration in AAA samples, mainly associated with CD45-positive cells, but not in normal aorta samples. We provide new data concerning 15-PGDH expression in human AAA, showing that 15-PGDH is upregulated in AAA and mainly expressed in infiltrating leukocytes. Our data suggest that microvasculature was not involved in PGE_2_ catabolism, reinforcing the potential role of microvasculature derived PGE_2_ in AAA-associated hypervascularization.

## Introduction

Abdominal aortic aneurysm (AAA) is a vascular degenerative disease with high morbidity and mortality in the aged population in industrialized countries and mortality rates associated with rupture of AAA are high [1]. AAA formation and rupture are closely accompanied by inflammation and neovascularization of the media layer both contributing to the progressive weakening and dilation of the vascular wall [2]. Prostaglandin (PG) E_2_ a prevalent inflammatory mediator in many tissues and inflammatory diseases has been involved in angiogenic processes in cancer and other pathologies.

Animal models and human studies indicate that PGE_2_ is involved in the pathogenesis of AAA [3–5]. PGE_2_ binds to four distinct E-prostanoid receptors (EP1–4) that belong to the family of seven transmembrane G protein-coupled receptors [6]. Biosynthesis of PGE_2_ begins with the release of arachidonic acid by phospholipases from the membrane phosphoglycerides, which is in turn oxidized to PGH_2_ by the action of cyclooxygenase (COX) [7,8]. PGH_2_ isomerizes to PGE_2_ by PGE-synthases (PGES). The microsomal isoform of PGES (mPGES-1) is inducible by proinflammatory cytokines and seems to be the main isoenzyme involved in PGE_2_ biosynthesis under inflammatory conditions [9–12]. COX-2/mPGES-1 is widely regarded as the major contributing enzymatic chain for PGE_2_ biosynthesis under pathological conditions.

Despite the relevance of angiogenesis in AAA, information concerning COX-2/mPGES-1 derived PGE_2_ in the AAA-associated hypervascularization is limited and restricted to leukocytes COX-2-derived PGE_2_ [3,13–15]. We recently reported data supporting the role of microvascular endothelial cells (MVEC) COX-2/mPGES-1/EP-4 axis on the AAA associated hypervascularization [16]. We confirmed previous reports showing that COX-2 is upregulated in AAA and we found that mPGES-1 expression was also increased in AAA [16]. Both COX-2 and mPGES-1 have been found to be expressed in MVEC, VSMC and infiltrating leukocytes in AAA [10,11,16,17]. Nevertheless, the relative contribution of vascular cells *versus* infiltrating leukocytes to the pool of active PGE_2_ in AAA remains unclear. Regarding PGE_2_-mediated angiogenesis, the relative cell contribution to the active pool of this PG and the expression profile of PGE-receptors is relevant to understand the role of MVEC in AAA development.

Levels of PGE_2_ are regulated by its biosynthesis to degradation ratio. The first enzyme involved in PG catabolism is NAD+-linked 15-hydroxyprostaglandin dehydrogenase (15-PGDH). PGs are rapidly metabolized by the initial oxidation of the 15(S)-hydroxyl group catalyzed by 15-PGDH followed by the reduction of the 13,14-double bond generating 13,14-dihydro-15-oxo-prostaglandins [18]. This enzyme has therefore been considered key to the biological inactivation of prostaglandins. 15-PGDH is widely distributed in various mammalian tissues, lung being one of the most active [19], and it has been viewed as a tumor suppressor in the field of cancer [20,21]. Nevertheless, its role in cardiovascular diseases, particularly in AAA, is unknown. The present study was undertaken to compare 15-PGDH expression in samples of aorta from AAA patients and healthy donors and to determine its cellular localization.

## Materials and Methods

### Patients

The study was approved by the Comité Ético de Investigación Clínica del Hospital Santa Creu i Sant Pau, approval number 10/009/1048. Written informed consent was obtained from each patient. The study conformed to the principles outlined in the Declaration of Helsinki. The inclusion criteria for this study were patients who underwent open repair for AAA with an atherosclerotic aneurysm and in whom an infrarenal aorta biopsy was taken during the intervention. The exclusion criteria were inadequate or no aortic biopsy, pseudoaneurysms, and infectious or inflammatory aneurysms. All patients underwent surgery at Hospital de la Santa Creu i Sant Pau (HSCSP).

### Tissue samples

Samples were obtained from the remaining mid-infrarenal aortic wall after exclusion and prosthetic replacement of AAA. Normal aortas (NA) were obtained from healthy aorta from multiorgan donors and samples were also taken from the mid-portion of the infrarenal abdominal aorta at organ harvest. When a luminal thrombus was present it was separated before the aorta biopsy was taken and aortic tissue was washed twice with cold phosphate buffered saline (PBS). A portion of each sample was placed in RNAlater solution (Qiagen GmbH, Hilden, Germany) and stored at 4°C for 24 hours before long-term storage at −80°C until further processing for RNA isolation. When possible a portion was fixed in formalin solution 10% (Sigma-Aldrich, Inc St Louis, MO) for 24 h and included in paraffin for immunohistochemical studies.

### Clinical data definitions

Risk factor definitions used in this study were: diabetes mellitus: glycated haemoglobin >5.8% or use of oral antidiabetic drugs or insulin; arterial hypertension: systolic blood pressure ≥140 mm Hg, diastolic blood pressure ≥80 mm Hg or use of antihypertensive medication; hyperlipidemia: a total cholesterol >6.2 mmol/L, LDL cholesterol >1.70 mmol/L or triglycerides >1.65 mmol/L; smokers: current smokers and ex-smokers stopped smoking <1 year; chronic occlusive pulmonary disease (COPD): FEV1/FVC<0.7; and renal insufficiency: estimated glomerular filtration rate (eGFR) ≤60 mL/min/1.73 m^2^ calculated using the Chronic Kidney Disease Epidemiology Collaboration (CKD-EPI) equation [22].

### Microvascular endothelial cells (MVEC) culture

MVEC were isolated from human adult foreskins using a previously described technique [17,23]. In brief, foreskins obtained from adult circumcisions were placed in PBS supplemented with penicillin 200 units/mL, streptomycin 200 μg/mL and amphotericin B 0.5 μg/mL (all from Biological Industries, Kibbutz Beit Haemek, Israel). Foreskins were cut into 3 mm squares and placed in PBS containing 0.3% trypsin and 1% EDTA at 37°C for 30 minutes. Segments were then washed several times with PBS, placed in a Petri dish in M199 containing 10% foetal bovine serum (FBS), and individually compressed with the side of a scalpel blade to express microvascular fragments. The microvascular segments were passed through a 150 μm stainless steel mesh and collected by centrifugation at 300xg for 15 minutes. MVEC were seeded on a gelatin-coated cell culture flask and cultured in medium MCDB131 with 20% FBS; L-glutamine 2 mmol/L, penicillin 200 units/mL, streptomycin 200 μg/ml, EGF 20 ng/mL and bFGF 5 ng/mL (all from Biological Industries). When cells reached confluence, they were purified with Dynabeads CD31 (Dynabeads, Invitrogen Dynal ASA, Oslo, Norway) following the manufacturer’s instructions. Flow cytometry and positive staining for CD31, platelet endothelial cell adhesion molecule-1 (PECAM-1) confirmed the purity of the cell population. MVEC were used in passage 3

### Aortic VSMC culture

Aortic human VSMC cultures were established by an explant procedure from multi-organic donor aortas as previously described [10,11]. The artery was longitudinally split and the endothelium was removed by gently scraping. The tissue was minced and allowed to adhere to the culture flask by incubation in a small amount of DMEM containing 10% FBS. VSMC were characterized by α-actin positive staining.

### Analysis of mRNA levels in the tissues and culture cells

Tissues were homogenized in the FastPrep-24 homogenizer and Lysing Matrix D tubes (MP Biomedicals, Solon, OH). RNA was extracted using Trizol (Invitrogen, Carlsbad, CA), following the manufacturer's instructions. The total RNA from cell cultures was extracted using Ultraspec (Biotecx Laboratories, Inc., Houston, TX, USA) according to the manufacturer’s instructions. cDNA was prepared by reverse transcription of 1 μg RNA using the High-Capacity cDNA Archive kit with random hexamers (Applied Biosystems, Foster City, CA). mRNA expression of the selected genes was studied by real-time PCR in an ABI Prism 7900HT using pre-designed validated assays (TaqMan Gene Expression Assays; Applied Biosystems) and universal thermal cycling parameters. Relative expression was expressed as transcript/β-actin ratios.

### Analysis of prostanoids in the Tissue-conditioned media

Tissue-conditioned media were obtained from approximately 150 mg of NA and AAA aorta fragments by incubating the tissues in 1 mL serum-free DMEM (Biological Industries,) in a cell incubator for 48 hours. The medium was then recovered and kept at -80°C until analysis. Released PGE_2_ and 6-oxo-PGF_1α_ (the stable metabolite of PGI_2_), were analyzed by EIA (Cayman Chemical, Ann Arbor, MI) following the manufacturer’s instructions. To evaluate levels of PGE_2_ metabolites (MPGE_2_) we used an enzyme immunoassay (EIA) kit that converts 13,14-hihydro-15-oxo metabolites of PGE_2_ into a single stable derivative (Cayman Chemical) following manufacturer’s instructions.

### Immunohistochemistry

Immunohistochemical studies were performed using a rabbit polyclonal antibody against 15-PGDH (diluted 1:150) from Cayman Chemical. Three-micrometer sections of paraffin-embedded tissue samples were stained in a Dako Autostainer Link 48 using the Dako EnVision Flex Kit. Diaminobenzidine was used as chromogen.

### Double-fluorescence immunostaining

For antigen co-localization studies, double-fluorescence immunostaining was performed using a sequential method. After deparaffinization and antigen retrieval, blocking for non-specific binding was performed at 4°C overnight. Appropriate concentrations of antibodies were then sequentially applied for 1 hour at room temperature, with PBS washing after each incubation. Slides were first incubated with anti-CD45, anti-CD68, CD20 or CD3 (IR751, IR7613, IR604 and IR503 without further dilution, Dako), followed by incubation with Alexa Fluor 594 goat-antimouse IgG (diluted 1/200; Life Technologies). Next, slides were incubated with the anti-15-PGDH antibody followed by incubation with Alexa Fluor 488 goat-antirabbit IgG (diluted 1/200; Life Technologies). As a negative control, sections were incubated omitting primary antibodies. Samples were then mounted with ProLong Gold antifade reagent with DAPI (Molecular Probes, Life Technologies Co, Eugene, OR). Images were obtained using an SP5 Leica confocal microscope.

### Statistical analysis

Sigma-Plot software was used for statistical analysis. All data regarding transcript levels are expressed relative to β-actin x1000. All quantitative data in this study were non-normally distributed. We used the Mann-Whitney Rank Sum Test to compare the two groups. The Pearson Product Moment Correlation was used to evaluate the association between continuous variables after Log10 transformation of data with non-normal distribution. A "P" value below 0.05 was considered significant.

## Results

**Fig 1:**
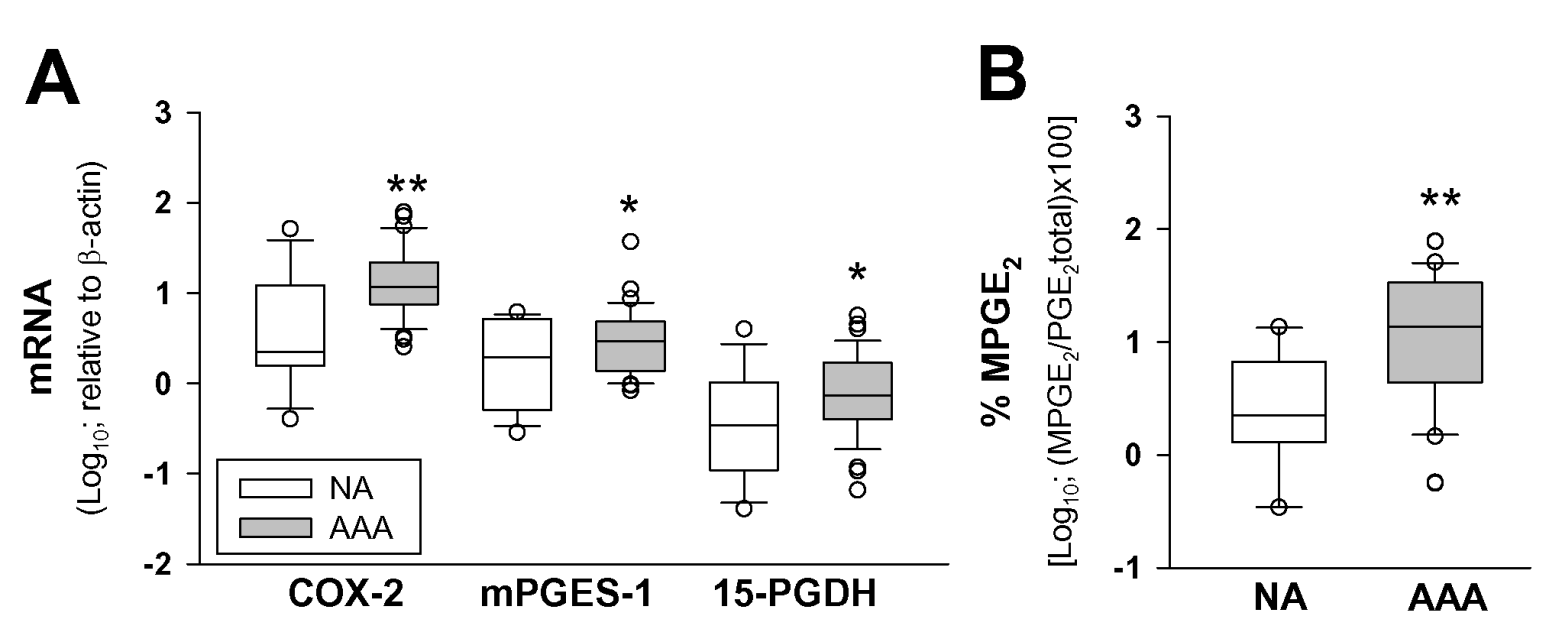
The expression of PGE_2_ biosynthetic and catabolic enzymes is increased in AAA. **A**, Box plot of expression levels of COX-2, mPGES-1 and 15-PGDH in controls (NA, n = 15) and abdominal aortic aneurysm (AAA, n = 39) samples; * p<0.05, when compared with NA samples. **B**, Box plot of the percentage of metabolized PGE_2_ (MPGE_2_) in NA (n = 12) and AAA (n = 30) samples; * p<0.01, when compared with NA samples.

Table 1 summarizes the characteristics of patients and donors included in this study. We analized the expression of 15-PGDH, COX-2 and mPGES-1 in AAA samples with NA by quantitative RT-PCR. The expression levels of any RNA transcripts did not fit a normal distribution. Results in Fig 1A show that, as expected [16], COX-2 and mPGES-1 expression was increased in AAA samples and that 15-PGDH mRNA was also significantly higher in AAA than in NA samples. Results regarding 15-PGDH were consistent with a significantly high ratio of metabolized PGE_2_ respect to total PGE_2_ in the tissue-conditioned media from AAA tissue samples than in NA (Fig 1B). As gender composition was different in AAA and NA populations, we tested for differences in the levels of 15-PGDH between males and females in the NA group (AAA group was 100% males) but found no statistically significant difference between genders

**Table 1:** Clinical characteristics of individuals with AAA and NA included in the study. Demographics and risk factors.

Measurements	mRNA		Secretion*	
Characteristic	AAA	NA	AAA	NA
Number	39	15	30	12
Aortic diameter (mm)	70.4±13.9	-	67.5±13.7	-
Age (years)	71.7±7.8	58.4±13.9	71.7±8.1	59.1±15.2
Male	39 (100%)	7 (46.7%)	30 (100%)	6 (50%)
Diabetes mellitus	7 (17.9%)	2 (13.3%)^a^	5 (16.7%)	2 (16.7%)
Hypertension	26 (66.7%)	2 (13.3%)^a^	21(70%)	1 (8.3%)
Hyperlipidemia	22 (56.4%)	2 (13.3%)^a^	20 (66.7)	1 (8.3%)
Smokers	10 (25.6%)	3 (20%)^a^	9 (30%)	2 (16.7)
Coronary artery disease	10 (25.6%)	0^a^	10 (33.3%)	0^a^
Chronic renal insufficiency	21 (53.8%)	0^a^	18 (60%)	0^a^
Peripheral vascular disease	19 (48.7%)	0^a^	16 (53.3%)	0^a^
Cerebrovascular disease	4 (10.3%)	1 (6.7%)^a^	2 (6.7%)	1 (8.3%)
COPD	7 (17.9%)	0^a^	7 (23.3%)	0^a^
Antiplatelet users	22 (56.4%)	1 (6.7%)^a^	14 (46.7%)	1 (8.3%))
Statins users	24 (61.5%)	0^a^	18 (60%)	0^a^
IECAs users	11 (28.2%)	0^a^	7 23.3%)	0^a^
NSAID users	2 (5.1%)	0^a^	3 (10%)	0^a^
Corticoid users	2 (5.1%)	0^a^	2 (6.7%)	0^a^
Immuno-suppressors users	1 (2.6%)	0^a^	1 (3.3%)	0^a^

Nominal variables are presented as number and as percentage (%) and continuous variables as mean±SD. Abbreviations: Aortic diameter: aneurysm maximum transverse diameter in mm. Chronic renal insufficiency: estimated glomerular filtration rate (eGFR) ≤60 mL/min/1.73 m^2^. Smokers: smoking in the last year. COPD: chronic obstructive pulmonary disease. Secretion*: prostanoid secretion by NA and AAA samples.

^a^ In some cases due to the nature of NA samples some of the clinical characteristics are not always recorded and infra-evaluation of them is probable.

**Fig 2:**
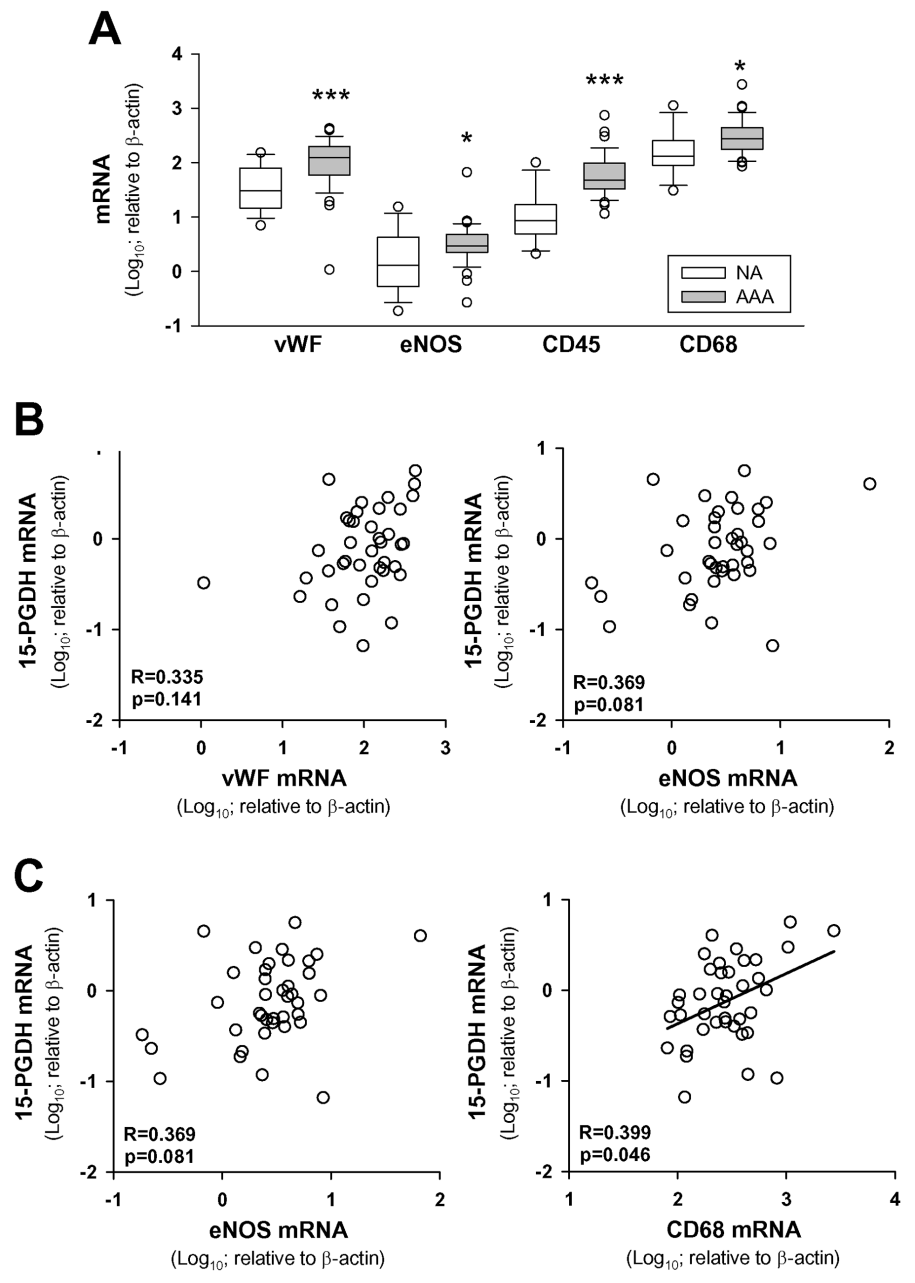
15-PGDH expression is associated with leukocyte markers in AAA. **A**, Box plot of expression levels of cell markers in controls (NA, n = 15) and abdominal aortic aneurysm (AAA, n = 39) samples; * p<0.05, *** p<0.001 when compared with NA samples. **B** Statistical correlations between transcript levels of 15-PGDH and endothelial cell markers in AAA samples; **C**, statistical correlations between transcript levels of 15-PGDH and leukocyte markers in AAA samples. Pearson Product Moment Correlation after logarithmic transformation of data and Bonferroni’s correction for multiple testing was applied; (n = 39.)

Attempting to ascertain if PGE_2_ degradation occurs in AAA microvessels, we determined the correlation between mRNA levels of 15-PGDH and those of MVEC and leukocyte markers. We analyzed the expression of the von Willebrand factor (vWF) and the endothelial nitric oxide synthase (eNOS) as endothelial cell markers and CD45 and CD68 as leukocyte markers. Cell markers analyzed were significantly enhanced in AAA samples (Fig 2A). Correlations between 15-PGDH and MVEC markers were not significant (Fig 2B), but 15-PGDH transcript levels were significantly associated with both leukocyte markers. The correlation of 15-PGDH with the macrophage marker CD68 was less significant than with the pan-leukocyte marker CD45 (Fig 2C).

**Fig 3:**
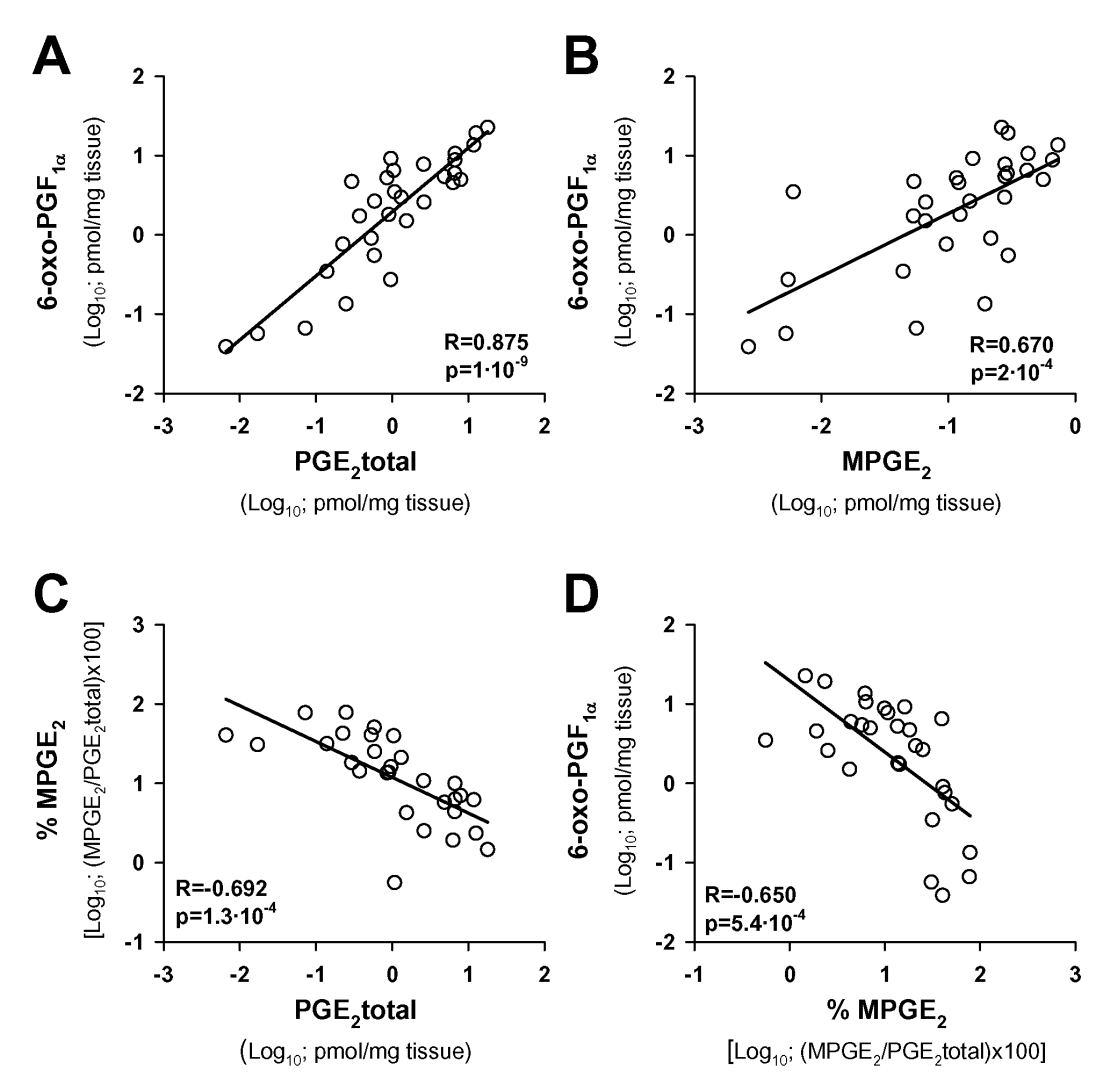
PGE_2_ production is associated with PGI_2_-producing cells. **A**, Statistical correlation between levels of 6-oxo-PGF_1α_ and total PGE_2_ secreted by AAA samples; **B**, statistical correlation between levels of 6-oxo-PGF_1α_ and PGE_2_ metabolites (MPGE_2_) secreted by AAA samples. **C**, Statistical correlation between the percentage of metabolized PGE_2_ secreted by AAA samples [%MPGE2; (MPGE_2_/totalPGE_2_)*100] and total PGE_2_; **D**, statistical correlation between secreted levels of 6-oxo-PGF_1α_ and %MPGE_2_. Pearson Product Moment Correlation after logarithmic transformation of data and Bonferroni’s correction for multiple testing was applied; (n = 30).

**Fig 4:**
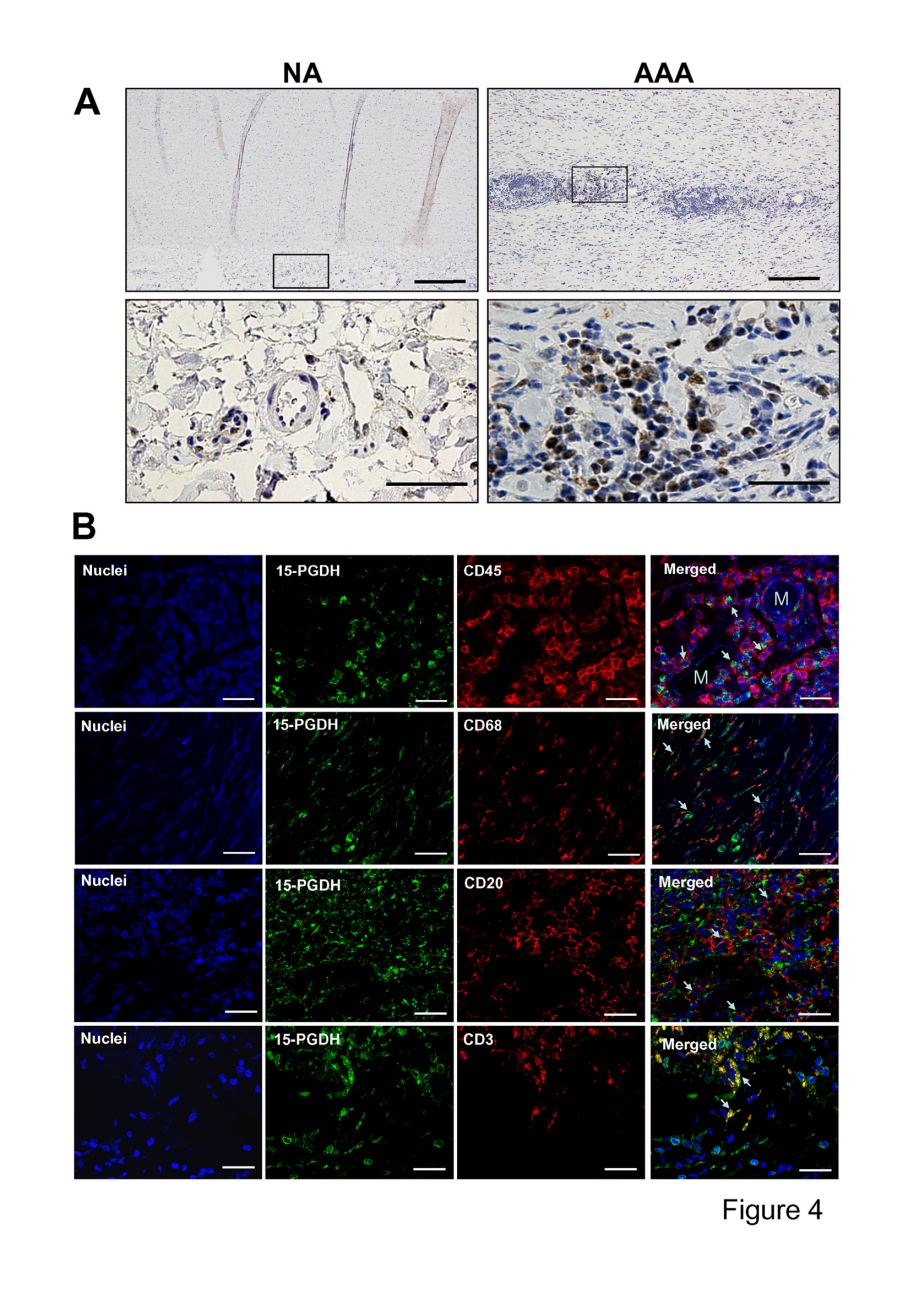
15-PGDH expression in AAA is mainly associated to CD45 cells. **A**, Representative immunohistochemistry images of 15-PGDH in NA and AAA samples, arrows show medial leukocyte immunostained with anti-15-PGDH; bars are 200μm (upper panels) and 50μm (lower panels). **B**, Representative immunofluorescent double staining for 15-PGDH and leukocyte markers in leukocyte infiltration areas; arrows show double immunostained cells; M indicates the light of microvessels; bars are 25μm.

In an attempt to approach the relative contribution of the endothelium to PGE_2_ production and degradation in AAA, we determined the statistical association of PGE_2_ levels with other cell-characteristic prostanoids secreted by AAA samples as independent variables. We evaluated PGE_2_, PGE_2_ metabolites (MPGE_2_) and 6-oxo-PGF_1α_, the stable metabolite of PGI_2_, which is not produced by leukocytes. As Fig 3A shows, a significant positive correlation was found between the production of PGE_2_ total and PGI_2_ (in terms of its stable metabolite) by AAA samples. We also observed a positive statistical association between levels of MPGE_2_ and PGI_2_ (Fig 3B). Nevertheless, the percentage of metabolized PGE_2_ showed a significant negative correlation with the total amount of PGE_2_ (Fig 3C) and with PGI_2_ (Fig 3D),

Our results indirectly indicated that PGE_2_ production was associated with PGI_2_-producing cells, and that degradation of PGE_2_ was negatively associated with the PGI_2_ production. We therefore performed immunohistochemistry to locate 15-PGDH in AAA and NA samples. Fig 4A show examples of the 15-PGDH immunostaining in NA and AAA samples. 15-PGDH protein was not found in NA samples and immunostaining in AAA samples was located in the areas of leukocyte infiltration. Immunofluorescent double staining showed that 15-PGDH was mainly associated with CD45-positive cells but also with cells expressing the macrophage marker CD68 (Fig 4B). To ascertain whether infiltrating lymphocytes accounted for 15-PGDH expression in AAA samples, we performed double immunofluorescence staining of the 15-PGDH and T-cell (CD3) and B-cell (CD20) markers. Results in Fig 4B show that 15-PGDH co-localize with both lymphocyte markers.

## Discussion

This study describes the expression of the 15-PGDH in human abdominal aortic aneurysm (AAA) for the first time. We found that 15-PGDH mRNA in aortic biopsies from AAA patients was significantly increased compared with aortic biopsies from healthy donors and its expression was mainly associated with infiltrating leukocytes. The MVEC COX-2/mPGES-1/EP-4 axis is relevant for PGE_2_-mediated hypervascularization from the early stages of human AAA development [16]. Here we confirmed our previous results [16] showing that expression of the PGE_2_-biosynthetic machinery, including COX isoenzymes and mPGES-1, is increased in human AAA. Nevertheless, the PGE_2_ degradation rate also contributes to this activity. 15-PGDH is the first enzyme responsible for the biological inactivation of PGE_2_ [19–21]. Here we found that 15-PGDH was upregulated in AAA, and consistently, the amount of MPGE_2_ secreted by AAA samples was higher than that secreted by NA. We previously reported that when patients were stratified according to the aortic diameter, the transcript levels of endothelial cell-markers and PGE_2_-biosynthetic enzymes were highest in the small diameter group (<55mm), while transcript levels of leukocyte markers were highest in the moderate diameter group (55–69.9mm) [16]. These observations led us to hypothesize that angiogenesis precedes the maximum inflammatory response during AAA development. Identifying the role of microvascular endothelium in PGE_2_-induced neovascularization in AAA, is a key question [2,23–25]. We therefore need to clarify whether the enhanced expression of 15-PGDH and hence PGE_2_ degradation occurs in microvessels or in the infiltrating immune cells.

In the present study, transcript levels of 15-PGDH were associated with the pan-leukocyte marker CD45 and also with the macrophage marker CD68, but to a lesser extent. These results indicate that 15-PGDH is associated not only with the macrophage population but also with other infiltrating leukocytes.

We confirmed our previous results showing that expression of COX-2 and mPGES-1 were increased in AAA [16]. COX-2 was found to be highly expressed in vascular cells and infiltrating leukocytes in AAA but weakly expressed in NA, suggesting that it was effectively up-regulated in vascular cells, including MVEC. We also observed that mPGES-1 was increased in AAA but the increase of mPGES-1 expression was not as high as that of COX-2 [16]. mPGES-1 immunoreactivity was located in all vascular cells including VSMC in both NA and AAA. In our previous work we proposed that a factor that could explain the modest increase of mPGES-1 found in AAA could be the breakdown of VSMC in AAA. Indeed, VSMC abundantly express mPGES-1 [10,11]. Our previous data indicated that both vascular cells and leukocytes potentially contribute to PGE_2_ biosynthesis [16]. The contribution of the different cell types present in aneurysmatic tissue to the PGE_2_ pool cannot be evaluated directly without seriously modifying tissue samples. In this work, we therefore used an indirect strategy to approach this matter. PGI-synthase is expressed in vascular cells—mainly in the endothelium—but not in leukocytes. We observed an excellent positive statistical association between the total secretion of PGE_2_ and PGI_2_ (in terms of 6-oxo-PGF_1α_) in aorta samples from AAA patients. This indicates that the biosynthesis of PGE_2_ in AAA would be associated with the cells producing PGI_2_ suggesting that MVEC contribute strongly to PGE_2_ biosynthesis in the AAA wall. Nevertheless, this circumstantial evidence should be corroborated by further studies. When we tested the statistical association between the percentage of metabolized PGE_2_ and total production of PGE_2_ we observed a negative significant correlation. As expected, similar results were obtained when we analyzed the association between levels of PGI_2_ and the percentage of metabolized PGE_2_ secreted to the incubation media. Moreover, we did not find 15-PGDH expression in terms of mRNA, either in MVEC or in aortic VSMC in culture (data not show). Altogether these results are consistent with the idea that PGE_2_ synthesized by the vascular endothelium is not inactivated *in situ*.

To confirm that 15-PGDH was mainly expressed in infiltrating leukocytes we performed immunohistochemistry and immunofluorescence studies. We did not observe 15-PGDH immunostaining in NA samples but analysis of AAA samples showed 15-PGDH immunostaining, mainly in the perivascular areas where the density of infiltrating leukocytes was highest [16]. The double immunofluorescence study showed coexpression of 15-PGDH with the leukocyte marker CD45, and to a lesser extent with the macrophage marker CD68, reinforcing the results obtained at mRNA level.

The presence of mPGES-1 in a particular cell is necessary for PGE_2_ biosynthesis [9–12,26,27]. In inflammatory diseases it is generally accepted that PGE_2_ comes from invading leukocytes, mainly macrophages. Macrophage COX-2-derived PGE_2_ is relevant in the pathogenesis and rupture of AAA [3,13–15]. Nevertheless, expression of 15-PGDH effectively regulates active PGE_2_ present in a particular cell type. We previously reported that MVEC express the PGE_2_ biosynthetic machinery [16,17]. We now add that 15-PGDH is expressed in leukocytes, thereby causing greater inactivation of PGE_2_ in these cells than in MVEC. Hypervascularization *per se* could be a determinant factor in reducing mechanical strength, because it turns the media layer spongy and favors leukocyte-mediated matrix degradation. We have shown that EP-4 activation, which is the main PGE-receptor in endothelial cells, induces angiogenesis [16,28]. Accordingly, MVEC-derived PGE_2_ could play key role in the AAA-associated hypervascularization since PGE_2_ could be more effective inducing angiogenesis in an autocrine manner.

In conclusion, we found that 15-PGDH, the first enzyme involved in PGE_2_ inactivation, is upregulated in AAA and its expression is mainly associated with infiltrating leukocytes. Considering that PGE_2_ plays a relevant role in angiogenesis, our results suggest that MVEC-derived PGE_2_ acts in the AAA-associated hypervascularization in an autocrine manner and that switching of endothelium to an inflammatory phenotype is a key point in AAA development.
